# Common Venues in Romantic Relationships of Adults With Symptoms of Autism and Attention Deficit/Hyperactivity Disorder

**DOI:** 10.3389/fpsyt.2021.593150

**Published:** 2021-06-18

**Authors:** Lorrayne Stephane Soares, Ana Luiza Costa Alves, Danielle de Souza Costa, Leandro Fernandes Malloy-Diniz, Jonas Jardim de Paula, Marco Aurélio Romano-Silva, Débora Marques de Miranda

**Affiliations:** ^1^Programa de Pós-Graduação em Medicina Molecular, Universidade Federal de Minas Gerais, Belo Horizonte, Brazil; ^2^Department of Mental Health, Universidade Federal de Minas, Belo Horizonte, Brazil; ^3^Department of Psychology, Faculdade de Ciências Médicas de Minas Gerais, Belo Horizonte, Brazil; ^4^Department of Pediatrics, Universidade Federal de Minas Gerais, Belo Horizonte, Brazil

**Keywords:** autism, ADHD, romantic (love), passionate love, attention deficit/hyperactivity disorder

## Abstract

**Introduction:** Autism Spectrum Disorder (ASD) and Attention Deficit/Hyperactivity Disorder (ADHD) figures among the most common neurodevelopmental disorders. Despite having opposite stereotypes, both ADHD and ASD compromise, though in different ways, skills such as social interactions, communication skills, and social thinking, which may underlie difficulties in romantic relationships.

**Methods:** We evaluated 306 adults about their romantic relationships and the intensity of their love. Participants were from one of four groups:, individuals with ASD-only traits, a group with symptoms of ADHD-only, participants with neither ADHD nor ASD symptoms (control) or from a fourth group of individuals with both ADHD and ASD traits.

**Results:** The ASD traits group had fewer married people and more people who have never been married, while the rate of divorce was higher in the ADHD traits group. Regarding the intensity of love, the mean scores of the ADHD and the ADHD+ASD traits groups were higher in the Passionate Love Scale than the mean score of the control group. Passionate love in the ASD group was no different from the other groups. Small positive correlations were found between passionate love and ADHD and ASD behavioral problems.

**Conclusion:** Marital status was distinct depending on symptoms of a neurodevelopmental disorder in adulthood. Also, ADHD and ASD symptoms were associated with greater passionate love. Therefore, ADHD and ASD behavioral dimensions may impact long-term and short-term experiences of a person's relationship with a significant other in distinct ways. Understanding how people with neurodevelopmental disorders experience love might help us to better clarify the mechanisms associated with their relationship patterns.

## Introduction

Neurodevelopmental disorders are childhood-onset conditions that commonly have a major impact on the personal, laboral, academic, and social functioning of individuals and their families. Two of the most frequent disorders diagnosed early in life are Autism Spectrum Disorders (ASD) and Attention Deficit Hyperactivity Disorder (ADHD), with a prevalence of ~1.8 and 5.29% of affected children (1, 2), respectively. Although these conditions are usually associated with childhood, 2.5–5% adults worldwide are affected by ADHD (3, 4), and 80–99% of all children with ASD diagnosis will meet criteria in adulthood (5, 6). Although ASD and ADHD show important differences in core symptoms, they share neurobiological, psychological, and behavioral characteristics, in addition to a high rate of co-occurrence (7–9).

ADHD is a heterogeneous disorder marked by inattentive and/or hyperactive/impulsive behaviors. Its functional impairment includes poor occupational performance, lower level of education, higher rates of delinquent behaviors, traffic accidents, self-reported happiness, marital dissatisfaction and divorce (1, 10–14). Although individuals with ADHD may often seem as outgoing and amusing people, they frequently suffer from social and interpersonal problems (15, 16). These social impairments encompasses a broad range manifestations as deficits in processing negative emotions in facial expressions or perceiving their inadequate behavior and inhibit ongoing actions toward more appropriate ones (17). They have poor social skills being commonly rejected by their peers and having difficulty to establish a relationship with a partner (15, 18). ASD shares some of the functional and social impairments associated with ADHD, such as difficulties in social interactions and communication (19). Impairment in social interaction is a core finding in ASD, coursing with difficulties to share interests, to understand and to describe facial expressions and feelings about yourself and others (20). ASD also is marked by a heterogeneous presentation, varying in cognitive disability, behavioral and functional profiles (21).

Considering that understanding, developing and maintaining intimate relationships requires social interactions, communication skills and social thinking (22, 23), individuals with ADHD and ASD might experience difficulties related to those (22, 24, 25). Indeed, Canu and Carlson (26) found that college students with the inattentive type of ADHD reported less and a later start of dating relationships, compared to college students with the combined type or with typical development. Girls diagnosed with ADHD in childhood reported fewer romantic relationships in late adolescence and early adulthood (27). Young adults with ADHD also presented an increased risk for undesirable pregnancies, higher frequency of sexually transmitted infections, casual sex and a greater number of sexual partners (28–32).

Concerning ASD individuals, data on their love life is inconsistent. Lau and Peterson (33) found that the presence of ASD symptoms didn't seem to influence marital satisfaction at a significant level, while Deguchi and Asakura (34) observe that wives of husbands with ASD report feeling lonely and socially excluded. Moreover, there is a general agreement that the frequency of ASD individuals in a romantic relationship is lower when compared with typically developed individuals (23).

Individuals with autism are frequently portrayed as insensitive and cold, that lack the need for love. Some traits, such as dislike being touched and poor comprehension of social signs, contribute to social stereotypes, labeling individuals with autism as asexual or uninterested in romantic relationships (22, 35–37), stereotypes which are reinforced by media which frequently consider autism as opposed to romantic love (38). However, researches indicate that adults with ASD would like to have romantic relations and report suffering from having those social impairments (39, 40).

Meanwhile, individuals with ADHD are often viewed as more creative, passionate and intense (12), a portrayal that is probably related to their high impulsivity. Acting without thinking or acting in the “heat of the moment” are very common behaviors among ADHD individuals, and such characteristics may contribute to describing them as passionate people who listen to their hearts or follow their instincts no matter what.

Nevertheless, these characteristics may also contribute to higher rates of divorce and poorer marital adjustment within adults with ADHD (41). Considering the response to undesirable partner's behavior, both the inattentive and the hyperactive/impulsive individuals present poor coping strategies to solve problems (42). Inattentive ADHD symptoms are related to less constructive reactions to the partners' failures and paying more attention to alternative partners. Those individuals presenting symptoms of hyperactivity-impulsivity present failure in inhibit negative responses to their partner and this could lead to the fail the search for an alternative partner.

Previous studies pointed to an association between marital quality and health, with divorce being correlated with poorer outcomes, as a greater risk for early death, for mental illness and with alterations in endocrine and immune function (43–45). Wherefore, passionate love, defined as a state of intense longing for union (46), a strong positive feeling toward the other one (47), seems to be an essential element. Although it is known as the first phase of a relationship, passionate love is important not only to initiate but also for maintaining a relationship, being predictive of relationship satisfaction, on both short and long-term relationships (48, 49). Love was also associated with relationship length. Results from Ratelle et al. (50) suggested that the stronger the feelings of love toward the partner, the greater the chances of the couple still being together after 3 months. Moreover, between other aspects, couples who divorced earlier showed lower levels of love when compared to couples who divorce later and with happily married couples (51).

Considering the impact on mental health and well-being of dysfunctional or unsatisfactory romantic relationships, our study aimed to investigate the characteristics of passionate love among individuals with symptoms of ASD and ADHD.

## Methods

### Participants and Procedures

Participants were 306 Brazilian adults (i.e., ≥18 years) recruited *via* the internet through a virtual snowball sampling strategy (banner ad posted at the researchers' social media) for a partially online open survey specifically designed to study cognition, behavior, and adaptive functioning depending on ASD traits in adults. The research is under the Helsinki Declaration principles and was approved by the local ethics board (registry: CAAE 56534516.1.0000.5149). Participants were assigned to four distinct groups according to clinical cut-off points on ADHD and ASD screening tools. To assess the ADHD symptoms we used the Adult Self-Report Scale (ASRS-v1.1) (52), and to evaluate autism traits we used the Autism-Spectrum Quotient (AQ) (53), besides a recall of behavioral symptoms' onset. Ninety-two (30%) participants had no criteria for clinical risk attribution (scores below the cut-off points and/or passed the age of onset), so they were grouped as typically developed individuals (i.e., control group). Forty-two (14%) participants were at clinical risk for ASD, 76 (25%) individuals for ADHD, and 96 (31%) showed scores suggesting a simultaneous risk for clinical ASD and ADHD (ASD + ADHD).

Participants voluntarily consented with their participation by clicking a button “I agree” in the electronic questionnaire after reading an invitation explaining the purpose of the study, the duration of the form, data that were being stored, and confidentiality security. No incentives were offered for voluntary participation. No technical issues were reported to our research team during data collection. Data were obtained from July 2016 to July 2017, and all questionnaire items were presented in a fixed order for all participants, independent of their response choices. Responses could be changed before submission, but not after. A second submission was not readily available for participants. Duplicates were identified by participants' full name, which was visible only for one researcher (JJP) before masking (each participant received a numeric ID), and only the first response was kept. Participants took an average of 30 min to respond to the electronic questionnaire.

### Instruments

#### Passionate Love Scale

To assess the passionate love intensity we used the short version of the Passionate Love Scale (PLS) (46), a 15-item instrument which, summed up, suggests a global measure on how much in love the respondent is. PLS is an unidimensional instrument, which evaluates cognitive, emotional and behavioral features of passionate love (46). The cognitive elements comprise the concerns related to the special other, as intrusive thinking and the partner or the relationship idealization. The behavioral and emotional features include aspects associated with the attraction toward the special one, as physiological arousal, physical proximity, positive and negative feelings, and availability to the other (46, 54, 55).

#### Sociodemographic Characteristics

The Brazilian Economic Classification Criteria (CCEB) was used to characterize the participants' socioeconomic status. Scores can vary from 0 to 100 and classified in one of six socioeconomic strata: A (monthly household income estimation of U$ 20888.00), B1 (monthly household income estimation of U$ 9254.00), B2 (average household income of U$ 4852.00), C1 (average household income of U$ 2705.00), C2 (average household income of U$ 1625.00), and DE (average household income of U$ 768.00) (56).

### Statistical Procedures

All analyses were performed with SPSS 22.0. Descriptive statistics and Spearman's correlation analyses were conducted within the entire sample to investigate variables distribution and their associations. Comparisons between the groups regarding passionate love, ADHD and ASD traits, instruction, sociodemographic measures and age, were performed by the Kruskal-Wallis test. Then, for further details of the results, we used Mann-Whitney tests (Bonferroni corrected) to specific group comparisons. Differences between groups regarding marital status and sex distribution were performed by chi-square tests.

## Results

**Figure 1:**
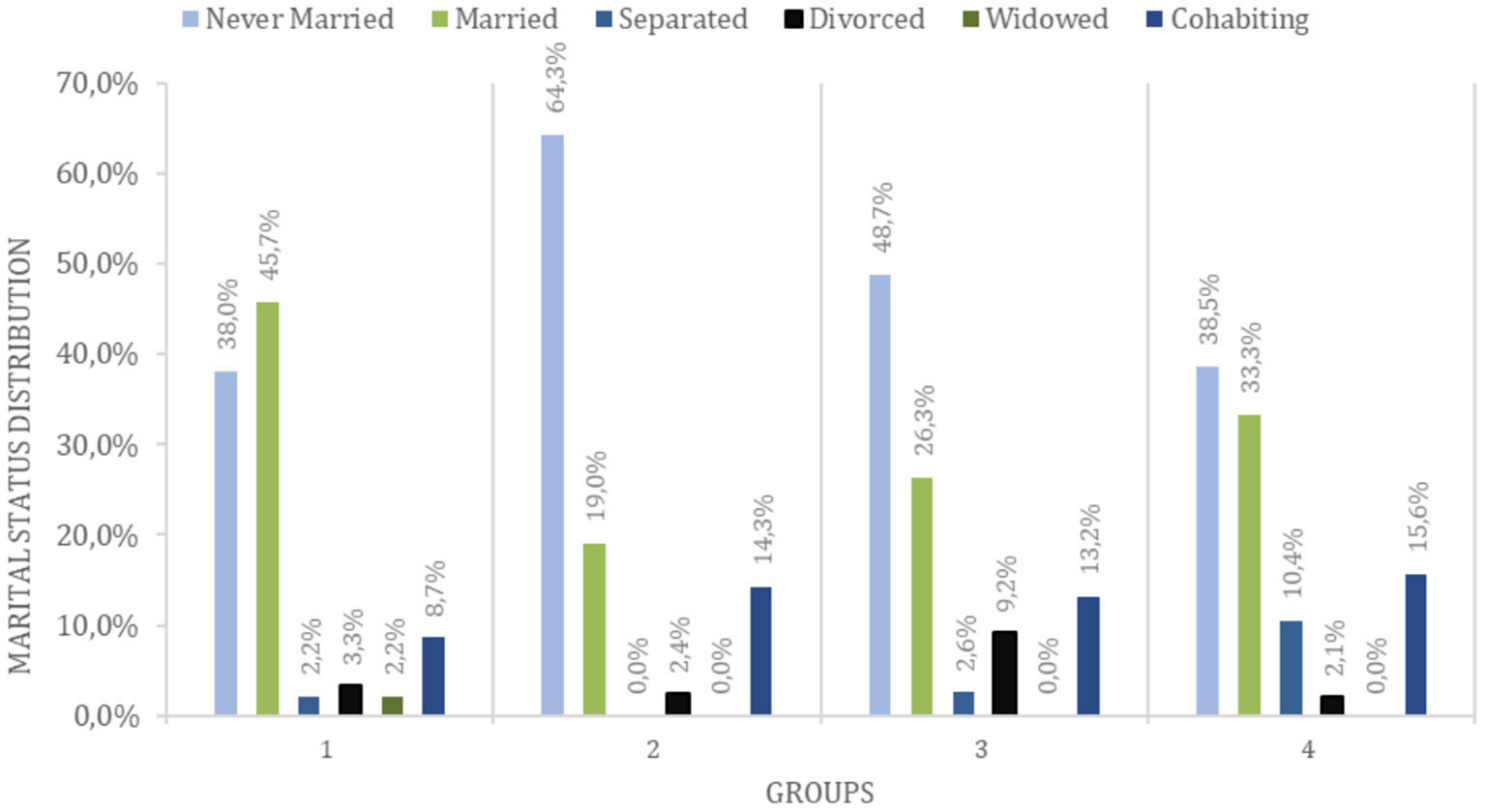
Marital status distribution by group. 1 = Control group, 2 = ASD-traits group, 3 =A DHD-traits group, 4 = ADHD + ASD-traits group.

In this survey, respondents' age varied from 18 to 58 years (*M* = 31.8; SD = 8.5) being predominantly female (73.9%). This distribution is in accordance with some results that suggest a gender bias, especially in surveys related to mental health and emotional issues (57, 58). Nevertheless, we found no significant differences in the distribution of sex (χ^2^ = 4.81 *p* = 0.186) between groups. The sample was classified according to the participants' scores on ADHD and ASD screening scales. Thus, 30.1% (Group 1, *n* = 92) had negative scores for both ADHD and ASD, 13.8% (Group 2, *n* = 42) had high scores for autism traits, 24.8% (Group 3, *n* = 76) had clinical scores only for ADHD, in which 34.2% were inattentive, 14.5% hyperactive, and 51.6% combined, and 31.4% (Group 4, *n* = 96) had clinical scores for both conditions. The participants' description is shown in Table 1. We found no significant differences between the distribution of age (*Z* = 2.44, *p* = 0.486), education (*Z* = 5.85, *p* = 0.119) and sociodemographic measures (*Z* = 0.68, *P* = 0.876) between groups. Of the total sample, 45.7% (*n* = 139) was in a steady relationship (married or living with their partner), while the others 54.3% (*n* = 165) had never been married, or were widowed, separated or divorced at the time. There were also significant differences in the marital status distribution (Table 2; χ^2^ = 36.948, *p* = 0.001) between groups (Figure 1 and Table 2).

**Table 1:** Participant's description and group comparison.

	**Control (*****n*** **= 92; 81.5% female)**		**ASD-traits (*****n*** **= 42; 66.7% female)**		**ADHD-traits (*****n*** **= 76; 75% female)**		**ADHD + ASD-traits (*****n*** **= 96; 69.8% female)**		**Group comparison**		
	***M*^a^**	**SD^b^**	***M***	**SD**	***M***	**SD**	***M***	**SD**	***Z*^c^**	***p***	Post-hoc^*^
Age	32.42	9.5	31.63	8.09	37.70	8.1	32.41	7.93	2.44	0.486	-
Education	20.63	5.73	19.43	5.35	20.20	6.5	18.81	5.65	5.85	0.119	-
Inattention (ASRS-18)	14.63	4.81	16.48	5.13	25.46	5.68	24.79	5.35	149.020	<0.01	1 < 3 > 2 < 4
Hyperactivity/impulsivity (ASRS-18)	13.7	5.61	15.38	4.93	21.74	5.93	22.23	5.57	105.346	<0.01	1 < 3 > 2 < 4
Autism quotient	20.79	7.0	37.83	3.29	22.57	5.98	38	4.07	227.598	<0.01	3 < 4 > 1 <2 < 4
Passionate love scale (15 items)	83.27	24.37	92.43	27.1	94.51	5.36	101.77	25.8	30.134	<0.01	3 > 1; 4 > 1

a *M, mean*.

b *SD, Standard Deviation*.

c *Z, Kruskal-Wallis-Test*.

* *Performed by group-group Mann-Whitney-tests with Bonferroni correction*.

**Table 2:** Marital status' distribution among groups.

	**Groups**			
**Marital status**	**Control (*n* = 92)**	**ASD-traits (*n* = 42)**	**ADHD-traits (*n* = 76)**	**ADHD + ASD-traits (*n* = 96)**
Married	42 (45.7%)	8 (19%)	20 (26.3%)	32 (33.3%)
Cohabiting	8 (8.5%)	6 (14.3%)	10 (13.2%)	15 (15.6%)
Never been married	35 (38%)	27 (64.3%)	37 (48.7%)	37 (38.5%)
Divorced	13.7 (3.3%)	1 (2.4%)	7 (9.2%)	2 (2.1%)
Separated	2 (2.2%)	0 (0%)	2 (2.6%)	10 (10.4%)
Widower	2 (2.2%)	1 (2.4%)	0 (0%)	2 (0.7%)

Regarding the intensity of passionate love, Table 3 shows a significant correlation with symptoms of inattention (*r* = 0.253, *p* < 0.01) and hyperactivity/impulsivity (*r* = 0.204, *p* < 0.01), assessed by ASRS-18, and with Autism Quotient (*r* = 0.212, *p* < 0.01) (Table 3). Comparing the groups' intensity of love, we found differences between the ADHD (*H* = −42.05, *p* = 0.013) and the ADHD + ASD-traits group (*H* = −70.55, *p* < 0.001) when compared with the control group (Table 1). Within the ADHD-traits group, we also found a significant difference, with a higher PLS score in the inattentive group (*Z* = −52.31, *p* = 0.001) and in the combined group (*Z* = −40.24, *p* = 0.007) when compared to the control group, while the hyperactive/impulsive group showed no significant difference (*p* = 0.058) in this comparison.

**Table 3:** Participants' correlations among passionate love and symptoms of autism and ADHD.

	**1**	**2**	**3**	**4**	**5**	**6**	**7**
1. PLS^a^	1.000	0.253^*^	0.204^*^	0.212^*^	0.103	0.049	−0.039
2. Inattention (ASRS-18^b^)		1.000	0.483^*^	0.234^*^	−0.075	−0.089	0.021
3. Hyperactivity/impulsivity (ASRS-18)			1,000	0.237^*^	−0.010	−0.010	−0.017
4. Autism quotient				1.000	0.077	−0.062	−0.087
5. Age					1.000	0.387^*^	0.218^*^
5. Education						1.000	0.286^*^
6. Sociodemographic status (CCEB^c^)							1.000

* *p < 0.01*.

a *PLS, Passionate Love Scale*.

b *ASRS-18, Adult Self-Report Scale*.

c *CCEB, Brazilian Economic Classification Criteria (higher scores suggest higher socioeconomic situation)*.

## Discussion

Previous studies have suggested that individuals with ADHD and ASD may experience difficulties related to romantic relationships, whether to initiate or maintain it (22, 25, 40). Previously, individuals with ASD have declared to have fewer romantic partners when compared to typically developed individuals (23). Individuals with ASD symptoms seem to have less exposition in any kind of relationship. In the ASD-traits group, there is a lower frequency of married people and a higher proportion of people who have never been married, while the ADHD appears to have a four times higher chance to have a divorce, confirming an impact in relationships. Both divorce and marital separation are two of the most stressful life events an individual can undergo, being considered more stressful than the death of a close relative or being in jail (59, 60).

Here we focused on the potential for any difference regarding the intensity of love feelings between people with high ADHD and autism traits and people without symptoms of any of these diagnoses. Romantic or passionate love is an intense emotional state typical of the beginning of romantic relationships, marked by profound feelings of attraction and commitment, as well as by obsessive characteristics, such as a jealous dependence and intrusive thoughts about the partner (48, 61). Some researches point to an association between romantic love, mental health and overall well-being and quality of life (48, 62), as well as with both marital and life satisfaction (63, 64). Moreover, if we exclude the obsessional aspects of early-stage love, it seems to be correlated with relationship satisfaction even in long-term relationships, suggesting that passionate love might be important not only in the formation but also in the maintenance phases of a relationship (48).

Our results suggest that the intensity of passionate love in ADHD-traits and the ADHD+ASD-traits group is greater than in the group with no symptoms, which could be interpreted as a trace of emotion dysregulation, a very common aspect among people with ADHD (65). Emotional regulation comprises a range of mechanisms associated with self-regulation, which encompasses some features as intensity, duration and stability of emotions, and skills of affection recognition, modulation and responsiveness (66–68). Although most researchers may focus on aspects related to emotional responsiveness, with special attention to reactivity to negative emotions, results from Rapport et al. (69) indicate that adults with ADHD appear to experience their own emotions with greater intensity, when compared to adults without the disorder, suggesting that individuals with ADHD may not only respond more intensely to their emotions but also feel that way.

Further analysis also suggested that the difference found in the ADHD-traits group was probably related to the inattentive dimension of the disorder. In previous studies, the ADHD inattentive presentation was associated with lower romantic satisfaction and less expression of love and affection (70). Indeed, behaviors that provoked the most negative reactions in partners of individuals with ADHD correspond to inattentive symptoms more than to hyperactivity (71). Moreover, inattentive individuals might be rated as unassertive and uninterested, which could explain their rejection rates regarding the beginning of romantic relationships (72).

These results are in accordance with the proposition that the point of difficulty in the interpersonal interaction might be in self-regulation of emotion as it is experienced and expressed (69). People with ADHD might have an accurate perception of social cues and their own emotions but may fail to act properly (65, 69), as individuals with ADHD seem to engage less in steady relationships and more likely to divorce, although they feel love more intensely.

The ASD-traits group does not differ from the ADHD, control and ADHD+ASD-traits group in the romantic love intensity, suggesting that adults with high autism traits do not experience less or more passionate love in their lives compared to the other groups. Although ASD has a very heterogeneous manifestation, difficulties with social interaction, in general, are a core trait, which includes deficits with verbal and non-verbal communication (21). They usually report difficulties expressing their feelings and emotions, to understand and predict other people's mental states and facial expressions (20). In addition, an individual with autism could have difficulties to initiate or to maintain a conversation, sharing their interests, or to modulate behavior considering the context (25, 73). Indeed, those aspects will impact the development of any kind of relationship, including romantic ones. A study developed by Stokes et al. (74), investigated the nature and predictors of social and romantic functioning in adolescents and adults with ASD. Their results suggested that the autism group reported less access to peers and friends compared to the control group, and this fact probably impacts the learning of social rules and romantic skills.

The common image of an individual with ASD is a person for whom love has no meaning, no draw, no neurochemical reward related to love, however, nothing of this picture was sustained by our data. Despite this, feeling in love is not a lacking emotion for those who have plenty of symptoms compatible with ASD. Our findings agreed with other reports, that individuals with autism, as well as those with a neurotypical development, also desire to be in an intimate and romantic relationship, even when they lack understanding of them, do not have skills, previous experiences, or knowledge to initiate those interactions (23, 75).

fMRI findings suggest that passionate love recruits brain regions involved in complex cognitive processing, such as social cognition, body image, self-representation and attention (76). We observe more or at least the same intensity of passionate love in those who have ASD and/or ADHD symptoms, but the mechanisms of ASD and ADHD symptoms impact in romantic relationships are still to be addressed in further studies.

An important limitation is the fact that we analyzed the relationship effects under the presence of symptoms without a diagnostic interview and evaluated only the romantic love effect without any information about the long-term features of love or less stable relationships. Another limitation was the survey's sex distribution, which was predominantly female. Since both ADHD and ASD are more prevalent among males, this could imply a sampling bias. Furthermore, there was also a difference in the sample distribution within the ADHD group, so the results should be interpreted with caution. A strength of our study was the evaluation of the dimensional effect presented in the individuals.

In conclusion, the relationship seems to be impacted by the presence of symptoms of neurodevelopmental disorders, having more impact on individuals with symptoms compatible with ASD. However, the impact apparently is not related to the intensity of love in the individual's point of view. Subjects with higher symptoms of ADHD and with high ADHD and ASD traits described having a higher intensity of romantic love, and nevertheless have less stable relationships. They are more likely to have a divorce, pointing toward a possible gap between feeling and expressing their emotions. Further studies are necessary to understand the mechanisms of differences and mechanisms of the relationship deficits. Improvement in relationships showed a beneficial impact and a universal protective factor, however, preventing bad relationships is a more important goal than getting an average relationship to a more satisfactory level (77). Interventions focused on preventing relationship dysfunction should have great potential. Thus, understanding how these population experiences love might help us to clarify points to be addressed in social training and mechanisms associated with their relationship patterns.

## Data Availability Statement

The raw data supporting the conclusions of this article will be made available by the authors, without undue reservation.

## Ethics Statement

The studies involving human participants were reviewed and approved by UFMG Ethical Board. The patients/participants provided their written informed consent to participate in this study.

## Author Contributions

Material preparation, data collection, and analysis were performed by LS and AA. The first draft of the manuscript was written by LS. All authors commented on previous versions of the manuscript, contributed to the study conception and design, and read and approved the final manuscript.

## Conflict of Interest

The authors declare that the research was conducted in the absence of any commercial or financial relationships that could be construed as a potential conflict of interest.
